# Early-Onset Osteoarthritis, Charcot-Marie-Tooth Like Neuropathy, Autoimmune Features, Multiple Arterial Aneurysms and Dissections: An Unrecognized and Life Threatening Condition

**DOI:** 10.1371/journal.pone.0096387

**Published:** 2014-05-07

**Authors:** Mélodie Aubart, Delphine Gobert, Fleur Aubart-Cohen, Delphine Detaint, Nadine Hanna, Hyacintha d’Indya, Janine-Sophie Lequintrec, Philippe Renard, Anne-Marie Vigneron, Philippe Dieudé, Jean-Pierre Laissy, Pierre Koch, Christine Muti, Joelle Roume, Veronica Cusin, Bernard Grandchamp, Laurent Gouya, Eric LeGuern, Thomas Papo, Catherine Boileau, Guillaume Jondeau

**Affiliations:** 1 INSERM U698, Hôpital Bichat, Paris, France; 2 AP-HP, Hôpital Bichat, Service de Médecine Interne, Hôpital Bichat, Paris, France; 3 AP-HP, Service de Médecine Interne, Hôpital Pitié-Salpétrière, Paris, France; 4 AP-HP, Hôpital Bichat, Centre de référence pour les syndromes de Marfan et apparentés, Service de Cardiologie, Paris, France; 5 AP-HP, Hôpital Bichat, Service de Cardiologie, Paris, France; 6 AP-HP Génétique moléculaire, Boulogne, France; 7 AP-HP, Hôpital Bichat, Service de Rhumatologie, Hôpital Bichat, Paris, France; 8 Université Denis Diderot, Paris 7, UFR de Médecine, Paris, France; 9 AP-HP, Hôpital Bichat, Service de Radiologie, Paris, France; 10 Université Versailles SQY, UFR des Sciences de la Santé, Guyancourt, France; 11 AP-HP, Département de Génétique, Hôpital Pitié-Salpétrière, Paris, France; University of Iowa Carver College of Medicine, United States of America

## Abstract

**Background:**

Severe osteoarthritis and thoracic aortic aneurysms have recently been associated with mutations in the *SMAD3* gene, but the full clinical spectrum is incompletely defined.

**Methods:**

All *SMAD3* gene mutation carriers coming to our centre and their families were investigated prospectively with a structured panel including standardized clinical workup, blood tests, total body computed tomography, joint X-rays. Electroneuromyography was performed in selected cases.

**Results:**

Thirty-four *SMAD3* gene mutation carriers coming to our centre were identified and 16 relatives were considered affected because of aortic surgery or sudden death (total 50 subjects). Aortic disease was present in 72%, complicated with aortic dissection, surgery or sudden death in 56% at a mean age of 45 years. Aneurysm or tortuosity of the neck arteries was present in 78%, other arteries were affected in 44%, including dissection of coronary artery. Overall, 95% of mutation carriers displayed either aortic or extra-aortic arterial disease. Acrocyanosis was also present in the majority of patients. Osteoarticular manifestations were recorded in all patients. Joint involvement could be severe requiring surgery in young patients, of unusual localization such as tarsus or shoulder, or mimicking crystalline arthropathy with fibrocartilage calcifications. Sixty eight percent of patients displayed neurological symptoms, and 9 suffered peripheral neuropathy. Electroneuromyography revealed an axonal motor and sensory neuropathy in 3 different families, very evocative of type II Charcot-Marie-Tooth (CMT2) disease, although none had mutations in the known CMT2 genes. Autoimmune features including Sjogren’s disease, rheumatoid arthritis, Hashimoto’s disease, or isolated autoantibodies- were found in 36% of patients.

**Interpretation:**

*SMAD3* gene mutations are associated with aortic dilatation and osteoarthritis, but also autoimmunity and peripheral neuropathy which mimics type II Charcot-Marie-Tooth.

## Introduction

Thoracic aortic aneurysms and dissections (TAAD) can occur as inherited mendelian diseases, appearing either as isolated events or associated within a spectrum of clinical features that define various syndromes. The oldest known syndrome is Marfan syndrome that associates TAAD with ocular alterations and other systemic features, listed in the recently modified Ghent nosology [1]. Marfan syndrome is mostly caused by mutations within the gene encoding fibrillin 1 (FBN1) [2], [3], the major component of connective tissue microfibrils. More recently TAAD were also linked to altered TGF-β signaling through the canonical Smad pathway. Indeed, the Loeys Dietz syndrome is linked with mutations within the genes encoding TGF-β receptors type I or II (*TGFBR1* or *TGFBR2)* [4]. Subsequently, mutations in the *SMAD3* [5] and *TGFB2* [6], [7] genes were identified. Mutations in the gene encoding Smad3 in autosomal dominant TAAD patients were recently associated with early onset osteoarthritis, defining a new entity: Aneurysms Osteoarthritis Syndrome (AOS) [5]. Such results were confirmed in subsequent reports [8], [9], [10], [11].

The objective of our study was to assess the full spectrum of clinical involvement in newly identified patients carrying a *SMAD3* gene mutation. Herein we report that mutations in this gene lead to an extended and more complex syndrome than previously recognized. The phenotypic features encompasses neurological alterations similar to those observed in the axonal type 2 form of Charcot-Marie-Tooth disease (CMT2) and include autoimmune manifestations.

## Materials and Methods

### Clinical Evaluation

All patients originated from the French National Reference Centre for Marfan Syndrome and related disorders. They were selected after gene screening showing that they carried a disease-causing mutation in the *SMAD3* gene. Family members were approached through the index case and invited to attend the National Reference Centre.

All patients initially had been evaluated by geneticists, rheumatologists or paediatricians (depending on their age), cardiologists and ophthalmologists to rule out syndromic forms of TAAD. Slit-lamp examination and cardiac ultrasonography were performed in all subjects. Once results of gene screening were available, all *SMAD3* gene mutation carriers were asked to undergo an extended workup provided they gave their written informed consent for participation in this clinical and genetic study in agreement with the requirements of French regulations (Accepted by “Comité de Protection des Personnes CPP Ile de France XI”, 78105 St Germain en Laye). For the purpose of the study and to ensure homogeneity, all retrospective clinical data were reassessed by one physician.

Comprehensive clinical examination used a structured evaluation form exploring each organ.

Laboratory tests included routine blood biology (hemogram, C reactive protein, prothrombin time, partial-thromboplastin time, sodium, potassium, chloride, calcium, phosphate, urea nitrogen, creatinin, aspartate aminotransferase, alanine aminotransferase, alkaline phosphatase, total bilirubin, lipase, lactate dehydrogenase, creatinin kinase, serum electrophoresis), autoantibodies (antinuclear antibodies and when positive anti DNA and anti nuclear soluble antigens; rheumatoid factor, anti citrullinated peptides, anticardiolipin and anti beta2 glycoprotein 1 antibodies), angiotensin converting enzyme, and urine testing for proteinuria.

Computed tomography (CT) of all arteries, including the aorta, with complete screening of the body including bone analysis. Two radiologists, one focusing on vascular abnormalities, the other looking at spinal alterations interpreted all the images.

Echocardiography was performed to measure aorta diameters at the level of the aortic root, the annulus, the sinuses of Valsalva, the sinotubular junction, the proximal ascending aorta, the aortic arch, the descending aorta, the abdominal aorta Aortic dilatation was considered when normalized diameters were greater than the mean plus 2 standard deviations, according to Roman criteria [12]. Mitral valve prolapse was defined by a coaptation occurring behind the mitral annulus in the parasternal long-axis view [13].

X-rays of both hands and wrists, ankles, knees and pelvis, were reviewed by rheumatologists.

Electroneuromyography (ENMG) was proposed for all individuals with clinical evidence for neuropathy. For each nerve, distal latency, conduction velocity, compound muscle action potential amplitude (baseline to negative peak), areas under negative phase and compound muscle action potential duration were measured. Sensory nerve action potential amplitude (peak to peak) was measured in the median, ulnar, superficial peroneal and sural nerves with surface recording and stimulating electrodes. Needle electromyographic detection study was performed in all patients.

### Classification of Patients

Probands and at risk relatives were called “mutation carriers” when a *SMAD3* gene mutation was demonstrated. At risk relatives who could not be screened for the presence of mutation were called “obligate mutation carriers” if either aortic dissection, aortic surgery or sudden death had occurred.

### Control Population

Hundred and ninety-eight consecutive subjects attending the National Reference Centre for Marfan Syndrome and related disorders from October to December 2011 (101 males, 97 females, median age 32 y.o., interquatile range 17–51 y.o., range 4–76 y.o.) were screened using a standardized questionnaire to evaluate prevalence of joint pain, cramps, paresthesia and Raynaud phenomenon and used as control according to final diagnosis (Marfan patients and normal subjects).

### Statistical Analysis

Qualitative variables were compared using Fisher’s exact Test, using R software. Odd ratios are reported using 95th confidence intervals.

## Results

Eight probands were identified in the data bank of the National Reference Centre for Marfan Syndrome and related disorders. They carry 8 never reported mutations in the *SMAD3* gene, located in exons 6 to 9 that encode the MH2 domain of the protein (Table 1). All these mutations affect highly conserved amino-acids and are predicted to be pathogenic by five computerized algorithms (PMut, http://mmb2.pcb.ub.es/PMut/; Polyphen, http://genetics.bwh.harvard.edu/pph/; SIFT, http://sift.jcvi.org/; SNPs3D, http://www.snps3d.org/; Mutation Taster http://www.mutationtaster.org/ *).* Family studies, when available, showed that no mutation was a *de novo* molecular event.

**Table 1:** Mutations identified in the *SMAD3* gene.

Famile	Exon	Nucleotide	Variation
BIC5301	Exon 6	c.733G>A	p.Gly245Arg
BIC9521	Exon 6	c.742T>C	p.Phe248Leu
BIC4191	Exon 6	c.860G>A	p.Arg287Gln
BIC886	Exon 6	c.668delC	p.Pro223Glnfs*18
BIC792	Exon 6	c.862_871+1dup11[AGACACATCGG]	p.Arg292Aspfs*53
BIC873	Exon 8	c.1102G>T	p.Arg368*
BIC029	Exon 9	c.1179_1180dupC	p.Cys394Leufs*4
BIC915	Exon 9	c.1267A>G	p.Ser423Gly

**Figure 1:**
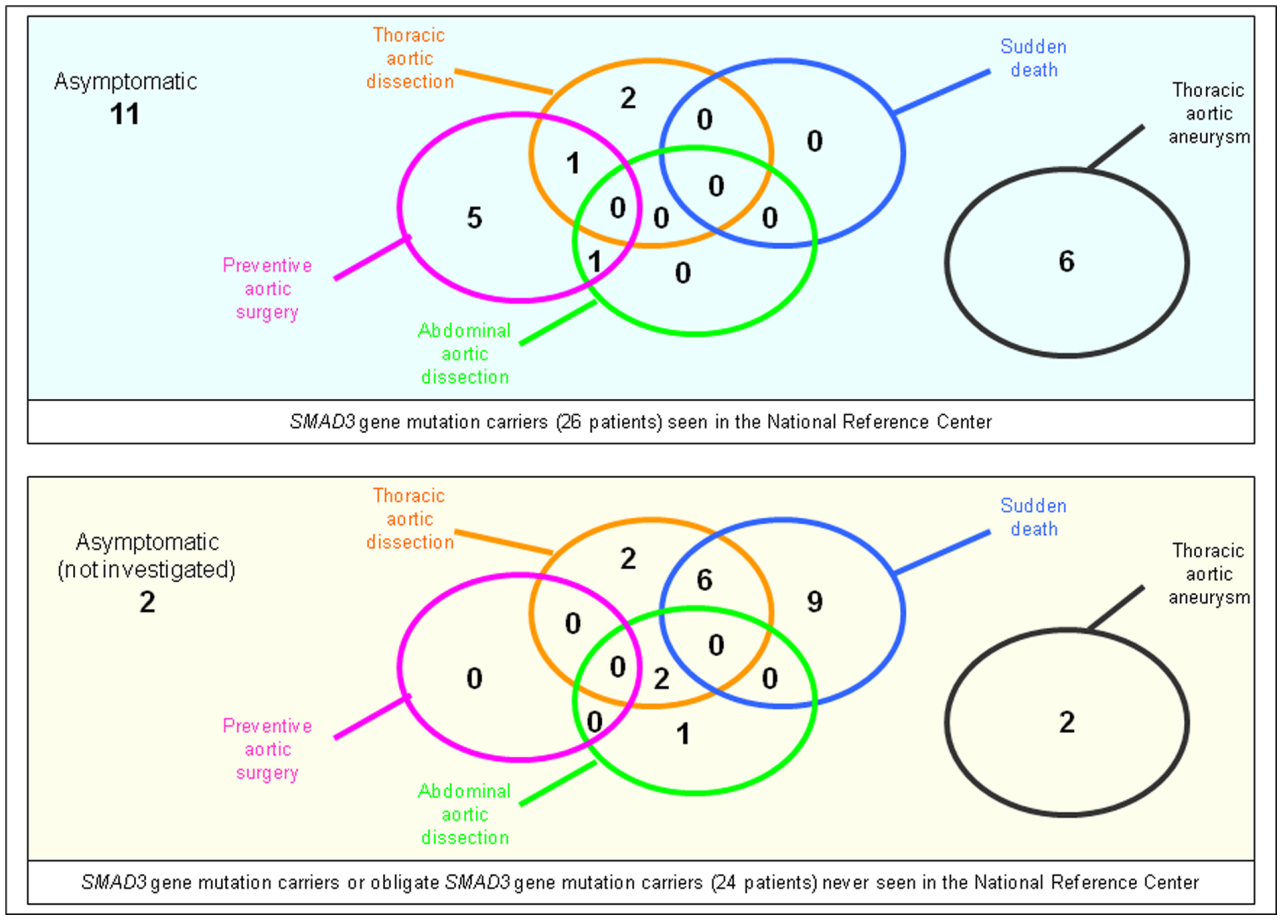
Description of the different patient populations, and explorations performed. ENMG: Electroneuromyogram, CT-scan: CT scanner.

Familial member recruitment allowed recognition of a total of 34 mutation carriers (17 males, 17 females, median age 52 years, interquartile range 37–60 y.o., range 8–83 y.o.), and additional 16 obligate mutation carriers (12 males, 4 females, median age 45 y.o., interquartiles range 33–51 y.o., range 22–68 y.o.), leading to a total population of 50 patients (Figure 1).

Previous testing in the National Reference Centre had previously been performed in 26 mutations carriers to rule out Marfan syndrome. In 23 of them (11 men, 10 women, 2 children) belonging to 6 unrelated families. additional extensive prospective medical evaluation could be performed (2 patients refused to come back within the delay of the study and 1 died between the two evaluations). This was completed by biological samples or data in 22, X-rays of peripheral joints in 14, and CT-scan of spinal TDM and aorta in 19 (not in 3 children and one pregnant woman.) ENMG was proposed to all patients with neurological examination abnormality, and performed in 9 who agreed.

### Cardio-Vascular Features

#### Aorta and mitral

**Figure 2:**
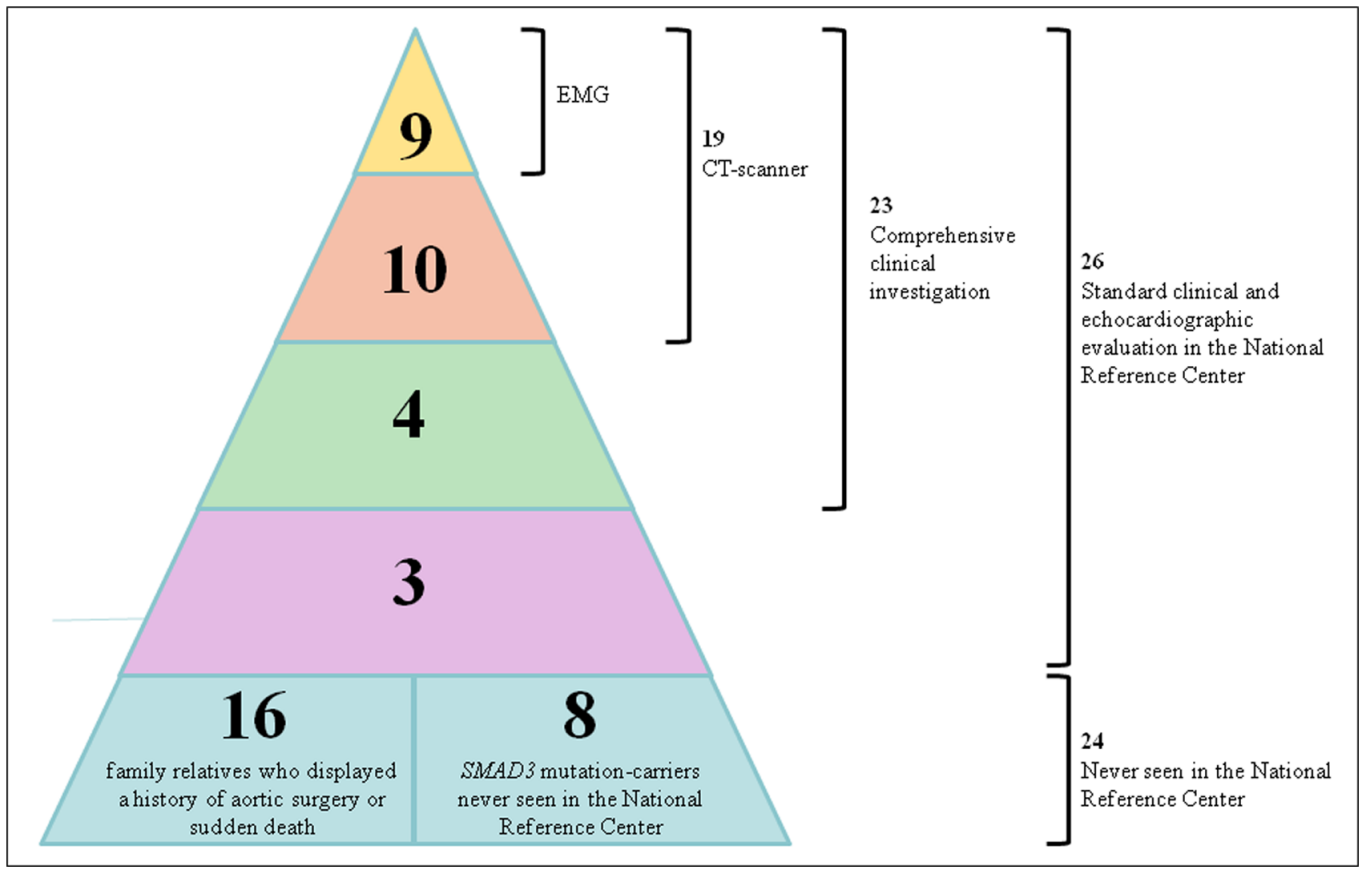
Aortic features in different subgroups. Abdominal aortic dissection is abdominal aortic dissection not related to thoracic aortic dissection extension. Asymptomatic subjects are patients without demonstration of any feature.

Aortic features were studied in all 50 subjects (26 mutation carriers and 24 obligate mutation carriers) (Figure 2). Disease of the thoracic ascending aorta was present in 36/50 (dilatation leading or not to surgery, aortic dissection or sudden death), and led to an aortic event in 28 (aortic dissection, preventive aortic surgery and/or sudden death) at a mean age of 44.8 years. Thoracic aortic dilatation was evidenced in 30% of the non-operated patients (8/26), maximal at the level of the sinuses of Valsalva. Four patients (8%, 4/50) had a history of abdominal aortic dissection, isolated in one patient. Overall, 54% (28/50) displayed documented aortic disease (aneurysm, dissection, surgery) and an additional 18% (9/50) sudden death of unknown origin (total 74% [37/50]).

Finally, mitral valve prolapse with mild mitral regurgitation was noted in 6 unoperated patients.

#### Other arteries and cardiac findings

Overall, 78% of the patients (14/18) had a dissection, aneurysm or tortuosity of neck arteries evidenced by a CT-scanner (Table 2). Furthermore, in 44% (8/18), involvement of other medium-sized arteries (subclavicular, renal, splenic or iliac) was diagnosed. All were asymptomatic, except for dissection of a coronary artery in one 58 years-old woman who suffered acute myocardial infarction. Interestingly, in this patient, CT-scanner also revealed multiple asymptomatic dissections of subclavian, carotid, vertebral, renal, and mesenteric arteries and no aortic dilatation. No cerebral artery aneurysm was found on CT scanner in our population. Overall, 95% (18/19) of the *SMAD3* mutation carriers who had been explored by CT-scanner displayed either aortic or extra-aortic vascular disease.

**Table 2:** Cardiovascular features.

	Percentage	Number	Mean age	Age range
***Disease of the ascending aorta***	**72%**	36/50	–	–
Uncomplicated dilatation >2 DS	30%	8/26	–	3–83
Preventive surgery	14%	7/50	46.0	27–58
Ascending aorta dissection	26%	13/50	46.6	30–68
Sudden death	30%	15/50	43.3	22–68
***Initial abdominal aorta dissection***	**8%**	4/50	46.3	35–56
***Disease of the neck arteries***	**78%**	14/18	–	–
**Carotid arteries**				
Dissection	0%	0/18	–	–
Aneurysm	33%	6/18	–	–
Tortuosity	72%	13/18	–	–
**Vertebral arteries**				
Dissection	6%	1/18	–	–
Aneurysm	22%	4/18	–	–
Tortuosity	22%	4/18	–	–
***Disease of other arteries***	**44%**	8/18	–	–
Dissection	6%	1/18	–	–
Aneurysm	39%	7/18	–	–
Tortuosity	11%	2/18	–	–

Furthermore, microvasculature was also involved. Raynaud phenomenon, with typical tricolor response, was found in 3/22 subjects (14%), while acrocyanosis (without pallor) was present in 11/22 patients (52%). No patient had digital ulcers. Since Raynaud phenomenon or acrocyanosis are frequent in the general population, we evaluated its prevalence in consecutive subjects attending the National Reference Center from October to December 2011 (Table 3). Prevalence of acrocyanosis was significantly greater in *SMAD3* gene mutation carriers as compared to healthy subjects (p = 0.005, OR = 6.7 [1.6–35.4]) or Marfan patients carrying a *FBN1* mutation (p = 4.10–5, OR = 9.12 [2.7–32.9]).

**Table 3:** Acrocyanosis, cramps and joint pains prevalence in *SMAD3* mutation-carriers and control patients (Cont).

	ACROCYANOSIS	CRAMPS	JOINT PAINS
**SMAD3 vs FBN1 (**2/129)	p = 4.30.10^−5^	p = 0.46	0.0055
Odd Ratio	9.12 [2.77; 32.91]		10.77 [3.62; 35.36]
**SMAD3 vs Cont (**22/32)	p = 0.0046	p = 0.067	p = 0.0023
Odd Ratio	6.71 [1.57; 35.41]		6.18 [1.68; 25.54]
**SMAD3 vs ALL (**22/198)	p = 1.02.10^−4^	p = 0.56	p = 0.0217
Odd Ratio	6.83 [2.41; 19.48]		2.47 [1.06; 5.68]

### Joint Disease

Osteoarticular manifestations were present in 100% of our population (Table 4). Pain was reported by 15 patients (mean age = 52 years) in peripheral joints, and by 17 patients (mean age = 44 years) in spine. Joint involvement was usually severe, requiring surgery in 9 patients at a mean age of 33 (ranging from 11 to 54 years-old) and involving the knee, ankle, wrist and/or spine. Spinal involvement was prominent, with X-ray or CT-scan proven osteoarthritis in 85% of patients (17/20), scoliosis (50%) or Scheuermann disease (22%). Peripheral joints were abnormal in 13 of the 14 available X- ray sets (93%), revealing classical lesions (knee osteoarthritis n = 9, rhizarthrosis n = 7, hip osteoarthritis n = 4) and more atypical features: osteoarthritis of unusual localization (tarsus n = 4, shoulder n = 2, carpus n = 2), lesions usually observed in crystal arthropathy such as narrowed scaphotrapezial joint space (n = 6), or calcification of the triangular fibrocartilage of the wrist (n = 2), and narrowed joint space of second metacarpal heads (n = 3).

**Table 4:** Rheumatologic manifestations.

**Clinical manifestations**		
Peripheral pain	70%	16/23
Peripheral deformity	22%	5/23
Hyperlaxity	17%	4/23
Sprain	39%	9/23
Spinal : cervical pain	17%	4/23
Spinal : lumbar pain	74%	17/23
**Radiological manifestations**		
Peripheral osteoarthritis	93%	13/14
Spinal osteoarthritis	85%	17/20
Scoliosis	50%	9/18
Osteochondritis	4%	1/23
Scheuerman	22%	2/9

Joint pain was significantly more frequent in *SMAD3* gene mutation carriers as compared to healthy subjects p = 0.002, OR 6.2 [1.7–25.5] or Marfan patients carrying a *FBN1* mutation (p = 0.006, OR = 4.3 [1.4–15.5]) (Table 3).

### Neurological Features

Overall, 68% (15/22) of patients belonging to 6 different families, displayed neurological symptoms such as muscle cramps, paresthesia, hypoesthesia, or gait disturbance (Table 3). Among these symptomatic patients, 9/15 belonging to 5 families had clinical evidence for peripheral neuropathy including absent tendon reflexes (n = 5), proprioceptive sensory loss (n = 1), recurrent sprain (n = 9) or pes cavus (n = 6). Three patients - belonging to 3 independent families – complained from severe paresthesia starting at adolescence. In the 3 patients, examination showed distal sensory loss and abolished tendon reflexes in lower limbs. All 3 patients had pes cavus and scoliosis. Electroneuromyography disclosed a pattern of axonal motor and sensory neuropathy in the 3 patients, including radicular denervation in 2 patients, in left S1 radicular nerve and in left L5 radicular nerve, respectively. Nerve conduction velocities were reduced in peroneal nerve with reduction of the compound muscle action potential amplitude and prolonged distal latency in two. Peroneal sensory nerve action potential was also decreased in all patients while sural sensory nerve action potential was decreased in two. Finally, compound muscle action potential amplitudes, distal latency and conduction velocity were normal in upper limbs in all patients (Table 5). In view of these results, ENMG was performed in 6 additional patients; only one displayed similar results. Hence, a total of 4/9 patients displayed a pattern of chronic sensory and motor neuropathy with decreased conduction velocity and compound muscle action potential amplitudes, consistent with CMT2-like neuropathy.

**Table 5:** Electroneuromyography in patients with severe neurologic symptoms.

	Patient 1	Patient 2	Patient 3
**Pes cavus**	Yes	Yes	No
**Scoliosis**	Yes	Yes	No
**Achille tendon reflex**	Not obtained	Not obtained	Normal
**Rotulian tendon reflex**	Not obtained	Not obtained	Normal
**Distal sensory loss**	Yes	Yes	Yes
**Vibration at the wrist**	Decreased	Normal	Decreased
**CMAP amplitude (peroneal)**	Decreased	Decreased (R)	Decreased (L)
R	1.39 mV	2.07 mV	5.91 mV
L	1.41 mV	6.04 mV	4.1 mV
**DL (peroneal)**	Limit	Prolonged (R)	Prolonged (L)
R	4.9 ms	5.6 ms	4.5 ms
L	4.9 ms	3.6 ms	5.6 ms
**CV (peroneal)**	Decreased	Decreased	Decreased
R	37 m/s	35 m/s	41 m/s
L	37 m/s	30 m/s	37 m/s
**CMAP amplitude (tibial post)**	Decreased	Normal	Normal
R	2.20 mV	6.77 mV	12.66 mV
L	0.81 mV	8.85 mV	13.65 mV
**Sural SNAP**	Decreased (L)	Decreased	Normal
R	13.6 µV	4.5 µV	17.4 µV
L	5.7 µV	5.7 µV	17.0 µV
**Peroneal SNAP**	Not obtained	Decreased	Decreased
R		2.3 µV	6.9 µV
L		3.3 µV	6.2 µV
**Upper limbs CV**	Normal	Normal	Normal
**Upper limbs CMAP**	Normal	Normal	Normal
**Upper limbs SNAP**	Normal	Normal	Normal
**Lower limbs detection**	Chronic neurogenic pattern	Normal	Normal
**Upper limbs detection**	Normal	Normal	Normal

The association of an axonal neuropathy with high arches and scoliosis was suggestive of axonal type 2 form of Charcot-Marie-Tooth disease (CMT2). CMT2 is a highly heterogeneous genetic disorder but 4 genes are the most frequently involved : the *GJB1* (connexin-32), *MPZ* (P0 or myelin protein-zero), *MFN2* (mitofusin 2) and *GDAP1* (ganglioside-induced differenciation-associated protein 1). To exclude a second gene defect, these genes were screened and no mutation was found. Conversely, *SMAD3* gene was screened in 70 patients with an autosomal dominant CMT2 but with no mutation in the major known CMT2-related genes (French National Reference Center, La Pitié Salpetrière hospital, neurology department). No mutation was found in the *SMAD3* gene in these patients.

### Autoimmunity

Eight patients (8/22 = 36%) presented with autoimmune features. One patient had primary Sjögren’s disease, defined by sicca syndrome, lymphocytic sialadenitis, anti Ro (SSA) antibodies, and numerous asymptomatic pulmonary cysts. One patient suffered clear-cut rheumatoid arthritis, with chronic arthritis in both hands and positive rheumatoid factor. Two patients - a 66 years-old woman treated for hypothyroidism and a 50 years-old man with prominent goitre with raised anti thyroperoxidase antibodies serum levels in both cases – suffered Hashimoto disease. Four patients had isolated – i.e. without clinical manifestations – autoantibodies: anti-nuclear antibody and anti- SSB/La, anti-citrullinated protein, anti-cardiolipin and anti-nuclear antibodies only, respectively.

### Allergy

Eleven patients (11/22 = 50%) displayed allergic manifestations such as asthma (n = 4), allergic rhinitis (n = 5), allergic conjunctivitis (n = 5), angiodema (n = 1), food allergy (n = 1) or eczema (n = 4).

### Marfan Criteria

No ectopia lentis was found among *SMAD3* gene mutation carriers. Skeletal features were heterogenous and Ghent 2 systemic scores ranged from 2 to 11.

## Discussion

We report on new findings in patients with *SMAD3* gene mutations that extend the clinical spectrum beyond aortic dilatation and osteoarthritis which were previously reported [5], [8], [9], [10], [11].

Neurological symptoms such as muscle cramps, paresthesia, hypoesthesia and gait disturbance were observed in the majority of *SMAD3* gene mutation carriers, half presenting an objective neuropathy. Three families out of 8 displayed a CMT2–like neuropathy on electromyography but none had mutations in the major genes usually responsible for CMT2. Because of the very low frequency (1/2500) of CMT2 in the general population [14], such results strongly suggest that *SMAD3* gene mutations are responsible for the CMT2-like phenotype observed in our patients. Of note, *SMAD3* gene mutations were not found in 70 CMT2 proband samples which were selected through a tertiary neurological centre. Interestingly, aorta imaging has not yet been performed in patients with CMT2 and no known specific mutations. Although TGF beta signalling may interfere with axon development [15] the pathogenic mechanism of neurological involvement in our patients is unknown.

We also observed auto-immune features in 36% (8/22) of patients, including Sjögren’s syndrome, rheumatoid arthritis, and Hashimoto disease. The role of the TGF beta pathway in the maintenance of peripheral tolerance in T-cells is well established [16]. Furthermore, transgenic mouse model with *Tgfbr2* conditional knock-out in dendritic cells develop multiorgan autoimmunity and premature thymus involution [17]. Hence, *SMAD3* gene mutations are expected to affect self-tolerance. Patients carrying mutations in other genes altering TGF beta signaling (*TGFBR1, TGFBR2, TGFB2*) may also warrant screening for autoimmune dysregulation [18].

Fifty percent of *SMAD3* mutation carriers suffered of allergic disease, especially asthma (23%) and allergic conjunctivitis (23%). Of note, a recent study suggested that mutations in genes encoding TGF beta receptor subunits may predispose to allergic disease [19].

In *SMAD3* mutation carriers, arterial disease is centered by TAA as an established predominant and life-threatening manifestation, observed in 72% of our patients (36/50). Neck arteries were also affected in 78% of the subjects (arterial tortuosity, dissection or aneurysm) and, unexpectedly, coronary and/or digestive arteries in 44% of the patients. In our cohort, CT-scanners of aorta and large arteries had a sensitivity of 95% (18/19) for diagnosis of the disease. Finally, Raynaud syndrome or acrocyanosis were observed in half of the *SMAD3* mutation carriers (significantly more frequently than in normal subjects or Marfan controls), suggesting also microvasculature involvement.

As expected from previous reports on *SMAD3* mutation carriers [5], [8], [9], [10], [11], we observed skeletal involvement in 100% of the subjects on X-ray or CT-scan study. Joint pain was significantly more frequent in *SMAD3* mutation carriers, as compared to both normal subjects or Marfan patients, even in the youngest patients. Joint involvement could be severe and treated with surgery in young patients, of unusual localization such as tarsus or shoulder, or mimicking crystalline arthropathy with calcifications and narrowed joint spaces. Because of the possibility of an underlying widespread vascular disease, we believe that aorta and arteries study should be discussed in patients suffering atypical osteoarthritis, either unusually severe, of early onset, or with atypical localization.

In conclusion, type 2 Charcot Marie Tooth -like neuropathy and autoimmunity may be added to the previously described atypical osteoarthritis and life-threatening aortic disease in the clinical spectrum associated with SMAD 3 gene mutation.
